# Machine Learning Assisted Classification of Cell Lines and Cell States on Quantitative Phase Images

**DOI:** 10.3390/cells10102587

**Published:** 2021-09-29

**Authors:** Andrey V. Belashov, Anna A. Zhikhoreva, Tatiana N. Belyaeva, Anna V. Salova, Elena S. Kornilova, Irina V. Semenova, Oleg S. Vasyutinskii

**Affiliations:** 1 Ioffe Institute, 26, Polytekhnicheskaya, 194021 St. Petersburg, Russia; anna_zhikhoreva@mail.ru (A.A.Z.); irina.semenova@mail.ioffe.ru (I.V.S.); osv@pms.ioffe.ru (O.S.V.); 2 Institute of Cytology of RAS, 4, Tikhoretsky pr., 194064 St. Petersburg, Russia; tatbelyaeva@gmail.com (T.N.B.); avsalova@gmail.com (A.V.S.); elena.kornilova@gmail.com (E.S.K.); 3 Institute for Biomedical Systems and Biotechnology, Peter the Great St. Petersburg Polytechnic University, 29, Polytekhnicheskaya, 195251 St. Petersburg, Russia

**Keywords:** digital holography, quantitative phase imaging, cell death, cell classification, HeLa, A549, 3T3, machine-learning algorithms, apoptosis, necrosis

## Abstract

In this report, we present implementation and validation of machine-learning classifiers for distinguishing between cell types (HeLa, A549, 3T3 cell lines) and states (live, necrosis, apoptosis) based on the analysis of optical parameters derived from cell phase images. Validation of the developed classifier shows the accuracy for distinguishing between the three cell types of about 93% and between different cell states of the same cell line of about 89%. In the field test of the developed algorithm, we demonstrate successful evaluation of the temporal dynamics of relative amounts of live, apoptotic and necrotic cells after photodynamic treatment at different doses.

## 1. Introduction

Machine-learning classification algorithms and neural networks have been demonstrated in recent years to be efficient tools for processing amplitude and phase distributions of wave fronts reconstructed using digital holography [1]. A number of papers have been published recently concerning applications of neural networks for reconstruction of extended depth-of-field images [2], cell detection and counting in diffraction patterns [3], adaptive aberration correction on phase images [4] or solution of autofocusing problem [5,6].

Neural networks were used as well for calculation of focusing quality metrics which allow one to find the best focused image in an image stack and accurately evaluate the distance between image and object planes [7]. Another method provides direct evaluation of focal distance from each reconstructed digital hologram, allowing for fast autofocusing without reconstruction of the image stack taken at various focusing distances [8]. Application of U-net-based architectures of convolutional neural networks can be used also for reconstruction of 3D particle distributions from digital holograms even at high particle concentrations [8]. The method has been extensively applied also for accurate cell segmentation on phase-contrast, fluorescence and differential-interference-contrast images [9,10].

Machine-learning approach can be successfully used in several routine tasks of data processing in digital holography, for example in aberration compensation [11], assistance in increase of spatial resolution by automatic stitching of data sets in synthetic aperture digital holography [12], tomographic reconstruction of three-dimensional distributions of refractive index [13] and identification of intracellular compartments during analysis of such distributions [14].

Machine-learning algorithms are increasingly applied in cytological research for analysis of various types of living cells using data obtained by digital holographic microscopy. Several works concerning classification of red blood cells using clustering algorithms have been reported. Complex analysis of 14 morphological features resulted in robust distinguishing between stomatocyte-, discocyte- and echinocyte-shaped red blood cells with as low as about 1% misclassification rate [15]. Another interesting example of application of convolutional networks is analysis of phase images of histological samples for their digital staining [16]. Although this approach can be used for cell segmentation on images obtained by fluorescent or wide-field microscopy as well [17,18], holographic microscopy often provides higher amount of quantitative data while being completely noninvasive for biological systems [19,20]. This makes the application of machine-learning algorithms for analysis of amplitude and phase images of living cells especially advantageous and promising.

In contrast with convolution neural networks machine-learning classifiers require preliminary evaluation of a set of specific features rather than direct analysis of phase images. Nevertheless, some machine-learning classification algorithms, such as support vector machines (SVM) algorithm, k-nearest-neighbor (k-NN) classifier or ensemble classifier (EC), allow for successful classifier training on labeled data and for sample classification within short computation time [21]. For example estimation of cell death time and distinguishing between apoptosis and primary lytic cell death was implemented using Long Short-term Memory neural network and SVM algorithm based on morphological parameters extracted from phase images [22]. In [23] low-coherent digital holographic microscopy was used for evaluation of 12 parameters of normal and cancer (both primary and metastatic) cells. Further analysis of the data obtained has shown statistically significant difference between these groups of cells in their major characteristics, and an automatic classifier was developed using SVM algorithm and principal component analysis for dimensionality reduction. Real-time detection of cancer cells in the blood flow in microfluidic channel using machine-learning classification algorithm with the accuracy of about 92% was recently demonstrated in [24]. Analysis of subcellular structures on automatically segmented quantitative phase images was also used for robust distinguishing between white blood cells and cancer cells [25]. Aside from investigation of cancer cells sperm cells at norm and under oxidative stress were classified using SVM algorithm on the base of 11 parameters obtained from quantitative phase images and used as predictor variables [26], malaria infected red blood cells were identified using multilevel ensemble classifier [27]. The combination of digital holographic recording and machine-learning classification algorithms was applied for identification of dead and living microalgae cells in the sea [28].

Quantitative phase imaging (QPI) techniques using coherent or partially coherent radiation [29,30] allow for reconstruction of phase images of individual cells and for computation of a wide variety of cellular parameters, discussed and listed in [27,31] and elsewhere. The obtained optical parameters of cells depend on both morphological characteristics and refractive index distributions, related to dry mass density in different cellular compartments. Analysis of these parameters allows for assessment of cell life cycle and growth [32], volumetry and refractometry [33], treatment efficacy of cancer cells [34,35], quantitative estimation of the dynamics of morphological changes [36,37], etc.

In this paper, we present implementation and validation of machine-learning cell death classification algorithm customized for three types of cell lines: HeLa, A549 and 3T3. Along with the robust identification of the cell type the algorithm allowed for distinguishing between different states of cells: live, necrosis and apoptosis. The developed classifier demonstrated accuracy of distinguishing between different cell lines of about 93% and of about 78% among cells of different lines and in different states (live, apoptotic, necrotic). The classifier operates with a database containing information on 10 optical parameters of individual cells, extracted from their phase images obtained using off-axis digital holography.

The paper is organized as follows. Section 2 contains the description of experimental approach and specifies a set of parameters needed for algorithm execution. In particular, in Section 2.1 we briefly describe our digital holographic microscope and data processing workflow for calculation of cellular parameters to be used for further classification, in Section 2.2 we evaluate the accuracy of estimation of cellular morphology from a single-phase image. Section 3 presents sample preparation protocol and cell treatment procedure inducing their death via different pathways. Section 4 describes the developed classification algorithm and its validation. The summary of the results obtained and conclusions made are given in the Conclusions section.

## 2. Digital Holographic Microscopy and Calculation of Cellular Parameters

### 2.1. Phase Image Reconstruction and Data Processing

Digital holographic microscope in the off-axis Mach-Zehnder layout with 20× microscopic objective (NA = 0.4) was used for acquisition of a set of off-axis digital holograms for each cell sample. Automatic recording of large sets of holograms for each sample was achieved using a motorized XY-translation stage (Standa) synchronized with a global-shutter Videoscan-205 CCD camera (Videoscan). Reconstruction of the recorded digital holograms was performed using a least square estimation algorithm with scanning area of 12 × 12 pixels [38,39]; minor phase aberrations were eliminated by subtracting a phase distribution corresponding to the absence of the object. Phase unwrapping using the Goldstein algorithm [40] and cell segmentation on the obtained phase images were performed afterwards for further calculation of cellular characteristics. The detailed description of the optical setup and workflow process can be found in our recent papers [37,41,42].

Several major parameters of individual cells were chosen for further analysis using cell classification algorithm. The list of parameters included [31]: (1) average phase shift $\varphi_{av}$, (2) cell phase volume, proportional to dry mass $V^{phase}$∼$DM^{cell}$, (3) projected area $S_{pr}$, (4) top phase surface area $S_{TopSurf}^{phase}$ (excluding attachment (projected) area), (5) phase membrane area $S_{membr}^{phase}$, (6) phase sphericity index $\psi =6\pi^{\frac{1}{3}}S_{membr}^{phase}/V^{phase}$ (7) variance $\sigma_{\varphi }$, (8) $Kurtosis_{\varphi }$ and (9) $Skewness_{\varphi }$ of phase shift distribution inside the cell Δφ and (10) cell eccentricity ε. A more general list of cellular parameters derivable from phase images, the corresponding equations and brief discussion can be found in [31].

Although the numerical processing of a 2D phase shift array induced by an individual cell allows for evaluation of a wider range of characteristics, which can be related to specific intracellular compartments and certain cell features, we believe that the chosen set of parameters carries information sufficient for robust classification of cells. Moreover, this set of parameters suggests somewhat of an ’integration’ of certain features over the entire cell area. This allows performing the robust analysis of cytological samples even in the case of low numerical aperture or non-negligible coherent and shot noise. Otherwise, detection of feature-specific characteristics (e.g., small apoptotic blebs or areas of efflux of intracellular medium during necrosis process) would necessitate high spatial resolution of the optical system and usage of additional techniques for coherent and shot noise suppression, which would make routine sample analysis more complicated or even impossible.

Furthermore, although all the selected parameters are, strictly speaking, related to optical characteristics of individual cells, some of them can also be interpreted as measures of morphological parameters under the assumption of a constant value of intracellular refractive index. We note that our algorithm operates solely with optical characteristics of cells obtained from their phase images. These data are robust and not affected by additional errors due to refractive index/thickness uncertainty. Further calculations of cellular morphology from the obtained data were performed for the sake of clarity. The developed classification algorithm was additionally validated on evaluation of the accuracy of cell morphology calculation from the analysis of phase images.

### 2.2. Evaluation of Cellular Morphology

The analysis of phase images of individual cells provides accurate data on their optical characteristics. Two-dimensional phase distributions recorded using digital holographic microscopy (DHM) depend on both spatial distributions of object thickness and refractive index. For obtaining data on morphological parameters of cells from their phase images this refractive index/thickness uncertainty should be eliminated. Despite local variations of intracellular refractive index, in some cases it can be assumed to be constant. This assumption allows for direct calculation of a height map of the cells and evaluation of their morphological parameters: average height $h_{av}$, volume *V* and membrane surface area $S_{surf}$. Variations of refractive index inside individual cells of the same type or among cells of different cell lines may lead to random and systematic errors in evaluation of cellular morphology. However, our research demonstrated that typical accuracy of cell morphology evaluation from DHM-assisted phase measurements is quite tolerable despite the assumption of constant refractive index of intracellular content.

**Figure 1:**
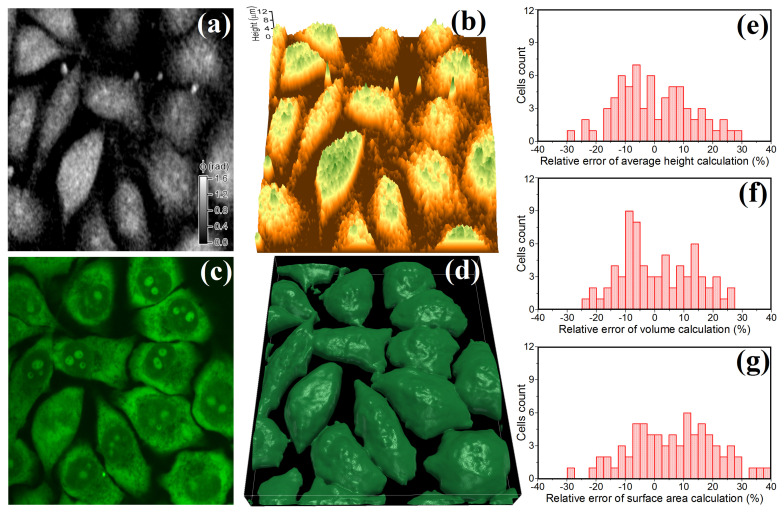
Examples of images of the same field of view in HeLa cell sample obtained by DHM and confocal fluorescence microscopy. (**a**) phase distribution obtained by DHM and (**b**) calculated height map; (**c**) XY-cross section image obtained using confocal fluorescence microscopy and (**d**) calculated 3D image. (**e**–**g**): statistical distributions of relative errors of average height $h_{av}$ (**e**), volume *V* (**f**) and surface area $S_{surf}$ (**g**).

To evaluate the accuracy of data on cellular morphology several fields of view within a sample of HeLa cells were monitored using both DHM and confocal fluorescence microscopy. Acquisition of several XY-cross section fluorescence images (Z-scanning step of ≈0.33 μm) was performed using Acridine Orange fluorescence dye which can bind to DNA and RNA in living cells. An example of fluorescence image is shown in Figure 1c. The analysis of the recorded stack of fluorescence images allowed for 3D reconstruction of cells (see Figure 1d) and determination of the morphological parameters $h_{av}$, *V* and $S_{surf}$ for several dozens of individual cells. The obtained cellular parameters were considered to be reference data for comparison with the characteristics retrieved from the analysis of phase images of the same cells. Assessment of morphological parameters of HeLa cells from their phase images (example shown in Figure 1a) was performed under the assumption of constant intracellular refractive index $n_{cell}=const$ after calculation of height distribution map $h\left(x,y\right)=\varphi \lambda /2\pi \left(n_{cell}-n_{medium}\right)$, where φ is phase shift, λ is recording light wavelength and $n_{medium}$ is refractive index of culture medium. An example of the obtained height distribution map in live HeLa cells is shown in Figure 1b. Relative calculation errors for average height $\delta h_{av}$, volume δV and membrane surface area $\delta S_{surf}$ were estimated for several dozens of individual cells in the sample. The obtained histograms of the relative errors of these parameters are shown in Figure 1e–g. The data obtained indicates that relative random errors of morphological parameters can be estimated as: δV=13.2%, $\delta h_{av}=12.9\%$ and $\delta S_{surf}=15.6\%$. Please note that due to presence of coherent noise and intracellular structures with high refractive index ripply surfaces can be observed on the height map, generated from phase images, which result in systematic overestimation of membrane surface area at about 7%.

Despite random errors arising in DHM-based morphology evaluation due to coherent noise, imperfect segmentation and variations of intracellular refractive index, we believe that the achieved accuracy was quite fair for analysis of individual cells and for distinguishing between cell types and states. Please note that the accuracy of cell morphology evaluation can be increased using high-NA microscopic objectives, using the synthetic aperture [43,44] and coherent noise suppression techniques [29,44,45]. The tolerable evaluation accuracy of morphological features provided by DHM indicates the validity of this technique for evaluation of morphology-dependent processes, such as necrosis/apoptosis cell death pathways.

## 3. Cell Samples and Photodynamic Treatment

### 3.1. Preparation of Cell Samples

The development and validation of cell classification algorithm was performed on the three established cell lines: HeLa (human cervix epidermoid carcinoma), A549 (human alveolar basal epithelial adenocarcinoma) and 3T3 (mouse embryonic fibroblasts). These specific cell lines are commonly used standard models and were chosen due to their wide usage in cytological research using QPI [46,47,48,49] and cytotoxicity assays [50,51,52,53,54] that are major potential applications of the developed classification algorithm. Even though these lines are of different metabolism and degree of malignancy, cancer (HeLa and A549) and pseudonormal (3T3), and represent different types of human and animal tissues. This diversity allows for validating the applicability of the developed approach for analysis of a wide range of cell lines. Cells were provided by the Russian Cell Culture Collection, Institute of Cytology RAS, St. Petersburg, Russia. Cells were cultivated in the Dulbecco’s modified Eagle medium (DMEM) supplemented with 10% fetal bovine serum and 1% penicillin-streptomycin at 37 ${}^{\circ }$C in 5% CO${}_{2}$ atmosphere.

Cell death was initiated by photodynamic treatment (PDTr) with Radachlorin photosensitizer (PS) (see Section 3.2 below for details). To provide PS accumulation in living cells they were incubated in 5 μg/mL PS solution in culture medium for 24 h. Noninvasive monitoring of cells by DHM was performed using CW HeNe laser operating at 633 nm with power density of about 50 μW/cm${}^{2}$ (note that the probe laser wavelength lay outside the PS absorption bands).

For evaluation of the accuracy of cell morphology determined using DHM, HeLa cells were seeded onto a cover glass and cultivated during 24 h. To reconstruct 3D morphology of cells by means of confocal fluorescence microscopy cells were stained by Acridine Orange (2.5 μg/mL) and Ethidium Bromide (5 μg/mL) for 1 min and several dozens of 3D fluorescence image stacks were recorded using confocal fluorescence microscope Leica TCS SP5 with ≈0.33 μm Z-axis scanning step. Then the cell sample was investigated by DHM.

### 3.2. Cell Death Induced by PDTr

Cell death via different pathways was induced in experiments by PDTr based on photosensitized intracellular generation of reactive oxygen species (ROS). The method uses light-induced excitation of PS molecules accumulated inside living cells. The clinically approved Radachlorin PS was used in experiments, which comprises a composition of sodium salts of chlorin e6 (≈80%), purpurin 5 (≈15%) and chlorin p6 (≈5%). Excitation of intracellular PS molecules was performed by a CW 660-nm laser radiation with power density on the sample varied from 10 mW/cm${}^{2}$ up to 140 mW/cm${}^{2}$. Samples of photosensitized cells were irradiated for 5 min and then monitored by DHM during 90 min.

The advantage of PDTr in induction of cell death is an opportunity for variation of cells response depending upon the treatment dose, as reported in [55,56,57]. Generation of ROS, including singlet oxygen molecules, may lead to programmed cell death through apoptosis pathway induced by activation of proteolytic caspase cascade, when the amount of generated oxidative molecules is moderate. In this case the programmed cell death prevents uncontrolled leakage of intracellular content to the environment and therefore prevents damage to neighboring cells and tissue inflammation [57]. On the contrary, high PDTr doses lead to generation of high amounts of ROS resulting in membrane rupture and necrosis process.

To identify the specific cells response at each of the applied irradiation dose standard biological tests were performed with Dead Cell Apoptosis Kit containing FITC Annexin-V and Propidium Iodide (Thermo Fisher Scientific), using a standard protocol for confocal fluorescence microscopy. Additional cell staining was performed using Acridine Orange and Ethidium Bromide (AO/EB) assay (Merck) for visualization of cells morphology and complementary verification of membrane integrity. The detection of cell death pathways in these reference experiments was performed using the confocal fluorescence microscope Leica TCS SP5 with 488-nm excitation wavelength. Fluorescence images were recorded in two spectral ranges: 500–560 nm for Annexin-V/Acridine Orange and 590–680 nm for Propidium Iodide/Ethidium Bromide. The apoptotic pathway of cell death triggered at low doses of PDTr was confirmed using the Annexin-V and Propidium Iodide (Annexin-V/PI) test assay, while cells necrosis was validated by observation of Propidium Iodide and Ethidium Bromide fluorescence of cells nuclei, indicating membrane rupture.

**Figure 2:**
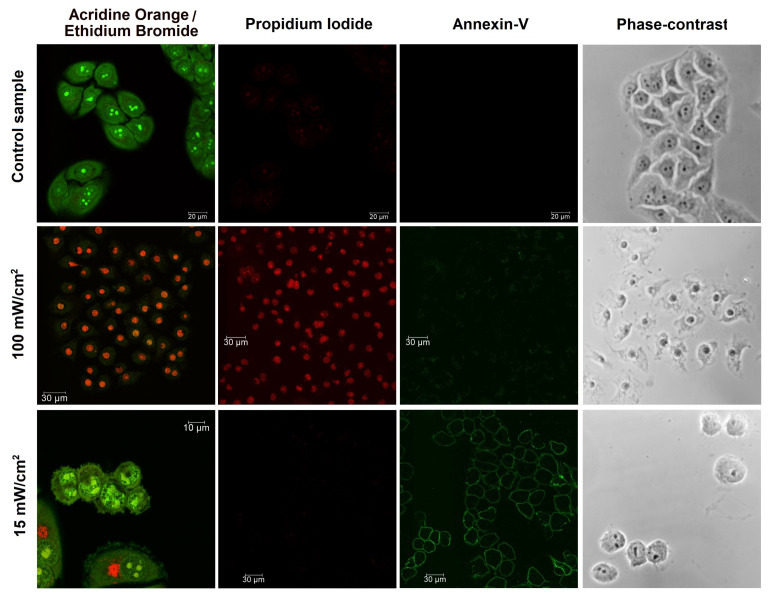
Examples of fluorescence and phase-contrast images of apoptotic and necrotic HeLa cells obtained after PDTr at different doses.

Two major pathways of cell death, apoptosis and necrosis, induced by PDTr in the three cell lines were studied at different power densities of excitation laser radiation. Apoptosis was induced by moderate power densities within the range of 10–46 mW/cm${}^{2}$. Please note that different cell lines demonstrated different resistance to PDTr, which manifested in different power densities required for inducing apoptosis in HeLa, A549 and 3T3 cells. The apoptotic death pathway was confirmed by strong Annexin-V fluorescence of cellular membranes in the green spectral range that allowed us to confirm the presence of phosphatidylserine on the outer leaflet of the plasma membrane, featuring initiation of the programmable apoptotic cell death. On the other hand, an absence of Propidium Iodide fluorescence in the red spectral range indicated integrity of cellular membranes. Necrosis was induced in the three cell lines using higher power densities of laser radiation within the range of 100–140 mW/cm${}^{2}$, providing rapid generation of ROS inducing membrane rupture and destruction of some intracellular compartments. The cells necrosis at these conditions was proved by red fluorescence of Propidium Iodide and Ethidium Bromide, both indicating membrane rupture through staining of cells nuclei. Typical fluorescence images of HeLa cells indicating apoptosis at lower irradiation dose and necrosis at higher dose are shown in Figure 2. Thus, the standard fluorescence tests allowed for determination of PDTr doses providing the two specific cell death pathways in the three cell lines. These data were used for analysis of the corresponding phase images of cells and development of a database for classifier algorithms. Phase images of cells of each of the three lines were divided into three groups: live, apoptotic and necrotic. Cells in the “live” group related to samples not subjected to PDTr.

## 4. Machine-Learning Based Cell Classification Algorithm

### 4.1. Development of Machine-Learning Cell Classification Algorithm

**Figure 3:**
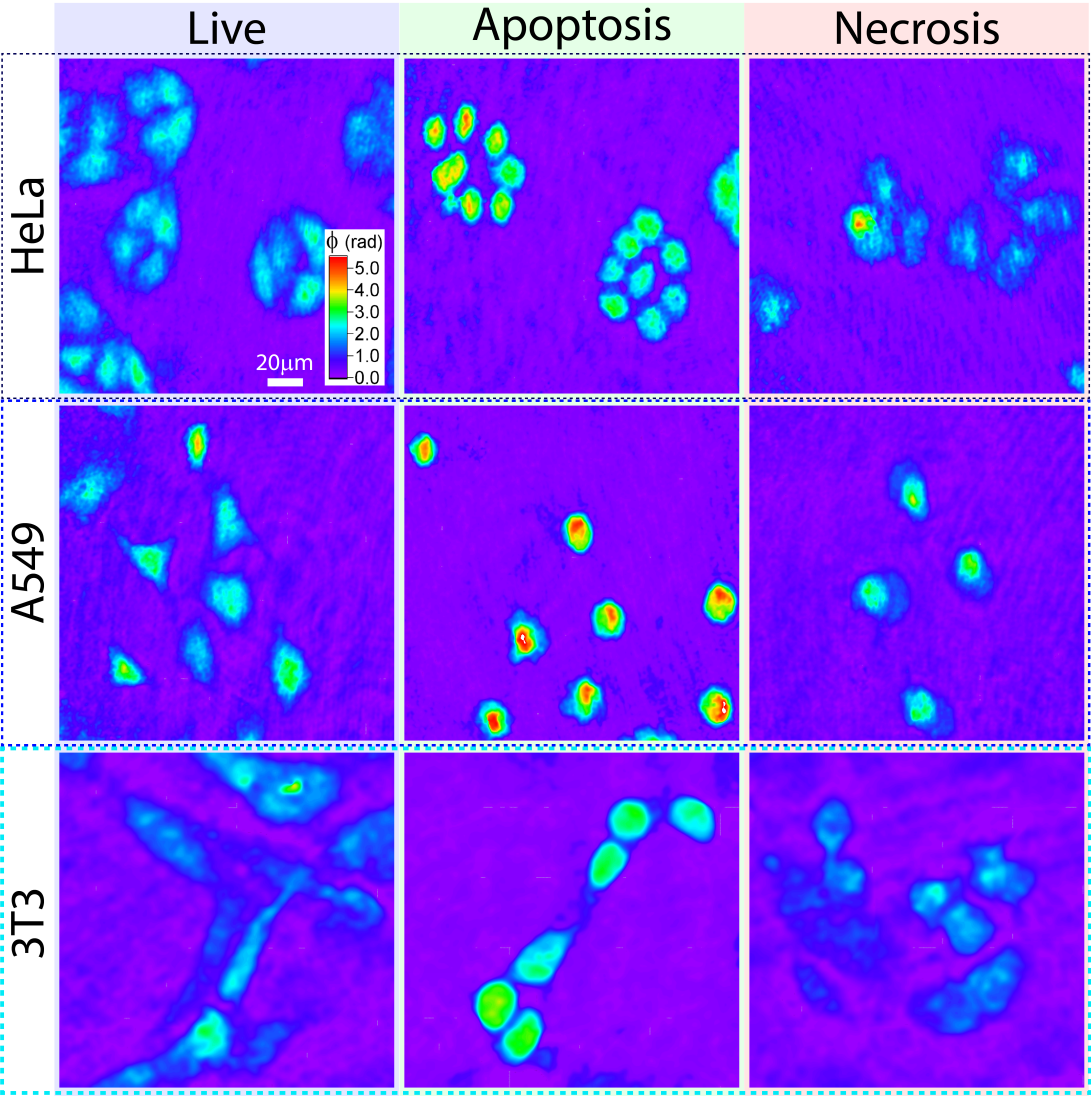
Examples of phase images of HeLa, A549 and 3T3 cells in three states: live, apoptotic and necrotic. An extended set of phase images of live HeLa, A549 and 3T3 cells is provided in Supplementary Materials for visual comparison of cells morphology.

To implement the cell classification algorithm the sets of digital holograms recorded during monitoring of 9 types of cell samples (3 above-mentioned groups of cells in each of the three cell lines) were analyzed. Typical phase images for each type are shown in Figure 3. In each type of samples phase images of no less than a hundred of individual cells were segmented and processed to evaluate 10 optical parameters listed in Section 2.1. The obtained database of retrieved cellular parameters corresponding to each type consisted of data on more than 1800 cells. The database was used for training of several classifiers, with 20% of the initial data set being used for evaluation of the classification accuracy. Three main types of machine-learning classifiers were applied for classification using the collected database: support vector machines (SVM) algorithm, k-nearest-neighbor (k-NN) classifier and ensemble classifier (EC). For each classifier several options have been tested, in particular: cubic, quadratic, linear, and Gaussian kernels in the SVM algorithm; cosine and cubic distance metrics, and distance weight in the k-NN algorithm; classifier that create ensembles (EC) of decision trees using AdaBoost algorithm, discriminant and nearest-neighbor algorithms. These methods were successfully applied for data processing in other studies related to holographic methods (see [58,59] and elsewhere). The most accurate option has been determined for each classifier and the data presented in Table 1 corresponds to the most accurate mode of each classifier.

Since classification was implemented basing on 10 parameters, the classifier algorithms involved an analysis of each data point in the 10-dimensional space. However not all the used parameters are equally useful for correct classification of cells; some of characteristics contribute more to the class identification. The analysis of the performance of implemented classifiers demonstrated that in general the most important parameters for classification are average phase shift $\varphi_{av}$, cell dry mass $DM^{cell}$, projected area $S_{pr}$, top phase surface area $S_{TopSurf}^{phase}$ and $Kurtosis_{\varphi }$ (in descending order of contribution to the classifier accuracy). For example, the classification among 3 lines of living cells with the SVM classifier demonstrated an increase of the classification accuracy from 50.2% (based solely on average phase shift as a predictor) to 78% and 92% in the cases of, respectively, 5 parameters listed above and all the 10 parameters.

**Figure 4:**
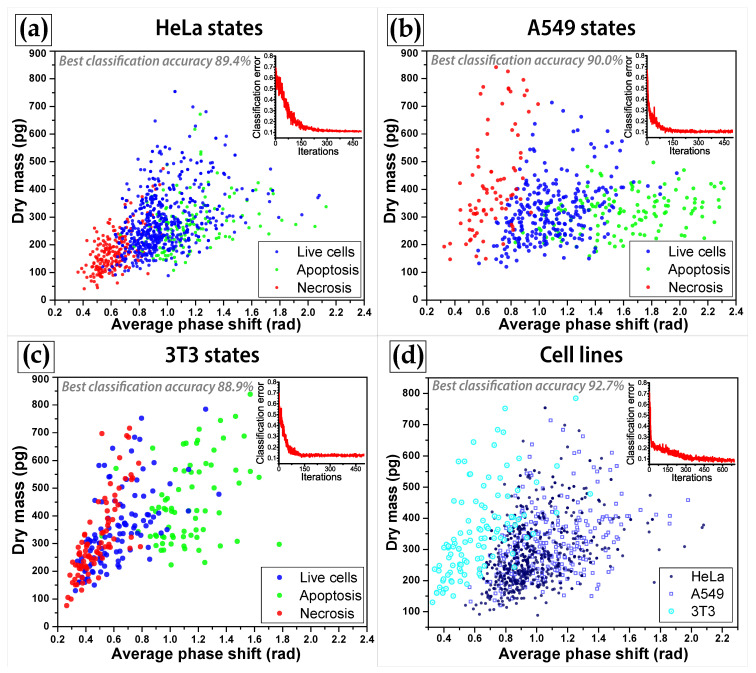
Scatter plots demonstrating classification of different states of HeLa (**a**), A549 (**b**), 3T3 (**c**) cells, and different cell lines (**d**) in the 2D space of average phase shift and dry mass. Please note that classification was actually based on 10 parameters of each cell. The classifier convergence curves are shown in the insets.

The classification of different cell lines was performed by means of three machine-learning classification algorithms. The 92.7%, 78.1% and 81.7% classification accuracy was achieved, respectively, by the SVM algorithm using quadratic kernel functions, distance weighted k-NN classifier and EC using Breiman’s random forest algorithm [60]. The same approaches were applied for the classification of living, apoptotic and necrotic cells within each cell line, HeLa, A549, and 3T3. The evaluated accuracy of the cell state classification ranged within 84–89% for HeLa, 82–90% for A549, and 83–89% for 3T3 (see Table 1). The highest accuracy was achieved for all cell lines by the SVM classifier. Algorithm performance over the entire database containing information on all the three states of three cell lines provided the accuracy of about 78% for SVM and about 67% for both k-NN and EC. Examples of scatter plots featuring cell differences within the two-dimensional space of average phase shift and dry mass are shown in Figure 4. We can conclude that the SVM classifier with quadratic kernel functions provided most accurate results on classification of both cell types and cell states.

To demonstrate the advantages of QPI for cell classification basing on their optical and morphological parameters the same classifiers were tested on the set of cell parameters which could be calculated from images obtained by bright-field microscopy. The list of cellular parameters calculated from segmented 2D amplitude images (without taking into account phase shift values) included: (1) cell projected area $S_{pr}$, (2) cell projected perimeter $P_{pr}$, (3) cell circularity (roundness calculated as $\left(4\pi S_{pr}\right)/P_{pr}^{2}$, (4) cell eccentricity ϵ, (5) cell solidity, (6) minor and major axes (see refs. [31,61,62] where some of these parameters were used for data analysis). Classification of cell states of the three cell lines using SVM, k-NN and EC algorithms basing on this set of parameters demonstrated classification accuracies within the ranges of 65.6–67.2%, 67.4–71.9% and 63.5–65.5% respectively. The accuracy of corresponding classification among cell lines comprised 64.9%, 60.1% and 59.7%. Please note that besides the significantly higher classification accuracy quantitative phase imaging allows for relatively easy cell segmentation using numerical algorithms that is not always the case for images obtained by bright-field microscopy.

### 4.2. Field Test of the Developed Algorithm

Thus trained classification algorithm based on the SVM classifier with quadratic kernel functions operating with the set of 10 cell parameters was applied to analysis of the dynamics of cell response to PDTr with different doses. As we have previously shown [36,42], different doses of PDTr, provided by variation of either irradiation power density or irradiation duration or both can cause different pathways of cell death and different death dynamics. In experiments we varied the power density keeping constant the irradiation duration. Measurements were performed on HeLa cell cultures.

**Figure 5:**
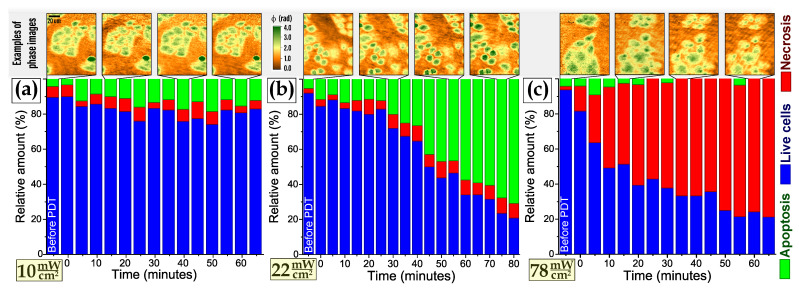
Relative amounts of live, apoptotic and necrotic HeLa cells after PDTr at different power densities of laser radiation calculated using cell classification algorithm based on SVM classifier: (**a**) 10 mW/cm${}^{2}$, (**b**) 22 mW/cm${}^{2}$, (**c**) 78 mW/cm${}^{2}$. First bar at each temporal dynamics represents cell states before irradiation. Time is counted from the end of PDTr. Examples of corresponding phase images obtained at different time moments are shown in the top row above each diagram.

In each measurement cycle monitoring of several dozens of fields of view was performed before irradiation of photosensitized cells and during at least 1 h after that. Phase images were recorded with a 5-min pitch. In the course of numerical processing of the recorded phase images several dozens of individual cells in each sample (typically 35–40 cells) were segmented and classified using the developed classification algorithm. Time-dependent variations of relative amounts of live, apoptotic and necrotic HeLa cells as classified based on phase image analysis are presented in Figure 5. Examples of phase images taken in the course of monitoring are shown above the histograms.

The obtained plots indicate significant difference in HeLa cells response to PDTr at different doses. At low dose (power density 10 mW/cm${}^{2}$) most cells observed in 60 min after PDTr were still classified as live cells (Figure 5a). Some slight growth of the number of apoptotic cells at low dose could be due to a specific response of HeLa cells with relatively low antioxidative protection. At slightly higher dose (22 mW/cm${}^{2}$) a prominent increase of relative number of apoptotic cells was observed starting at 20–25 min after PDTr (Figure 5b). Some slight increase of the number of necrotic cells was also detected. High power density of laser radiation (78 mW/cm${}^{2}$) resulted in predominant cell death through the necrotic pathway and rapid increase of the number of necrotic cells (Figure 5c).

Please note that due to imperfect cells classification not all the cells before PDTr were classified as ‘live’ cells. Besides slightly inaccurate classification (classification accuracy was below 100%) some variations of the amount of live, apoptotic and necrotic cells at different time moments in a sample were because at each time moment different groups of cells were studied. It is also interesting to note that high power density of laser radiation resulted in relatively rapid grow of necrotic cells portion, while the major increase of the fraction of apoptotic cells started only in 20–25 min after PDTr, which is apparently due to the programmed and energy-dependent nature of this process, requiring some time to proceed.

The results obtained are in a good agreement with the predominant cell death pathways, demonstrated using confocal fluorescence microscopy at similar doses of PDTr and our previous findings on HeLa cells resistance to PDTr with Radachlorin photosensitizer [37,41,42]. Therefore, the developed classification algorithm allows for robust distinguishing between different cell states and types.

## 5. Conclusions

We have presented the development and validation of machine-learning-assisted cell classification algorithm based on the analysis of their phase images, obtained using off-axis digital holographic microscopy. It was shown that the automatic analysis of 10 optical parameters, calculated from cell phase images allows for distinguishing between different cell types and different cell states (live, apoptotic and necrotic). The well-defined relation between some optical and morphological parameters of cells allowed for determination of cellular morphology despite variations of intracellular refractive index. The most robust classification algorithm based on analysis of optical parameters was found to be the support vector machine (SVM) algorithm with quadratic kernel functions. It allowed for distinguishing between different cell types with the accuracy of about 93%, and between different states of cells of the same line of about 89%. Moreover, the analysis of 10 optical parameters allowed for classification among cells of both different lines and states with the classification accuracy over 77%, which is quite a good result taking into account simultaneous distinguishing among 9 classes of cells. Moreover, the comparison of cell classifiers based on the data obtained using quantitative phase imaging and bright-filed microscopy demonstrated significant improvement of classification performance when optical parameters, calculated from phase images are taken into account. Validation of the developed classification algorithm on monitoring of the dynamics of cells response to PDTr at different irradiation doses demonstrated successful evaluation of relative amounts of live, necrotic and apoptotic cells.

## Figures and Tables

**Table 1:** Classification accuracy of states of HeLa, A549, and 3T3 cells, cell lines and states of cells among three cell lines using SVM (support vector machine), k-NN (k-nearest neighbors) and EC (ensemble classifier) machine-learning algorithms.

Classification between	SVM	k-NN	EC
Hela states	89.4%	84.4%	85.0%
A549 states	90.0%	89.0%	82.0%
3T3 states	88.9%	82.7%	83.9%
cell lines	92.7%	78.1%	81.7%
cell states in three cell lines	77.8%	66.5%	66.9%

## Data Availability

Data is available within the paper and in the supplementary materials.

## Supplementary Materials

The following are available online at https://www.mdpi.com/article/10.3390/cells10102587/s1.
