# Supplementary figures and images for: Machine Learning Assisted Classification of Cell Lines and Cell States on Quantitative Phase Images
Source: Cells. 2021 Sep 29;10(10):2587. doi: 10.3390/cells10102587 (PMC8533984; doi:10.3390/cells10102587)

HeLa live cells

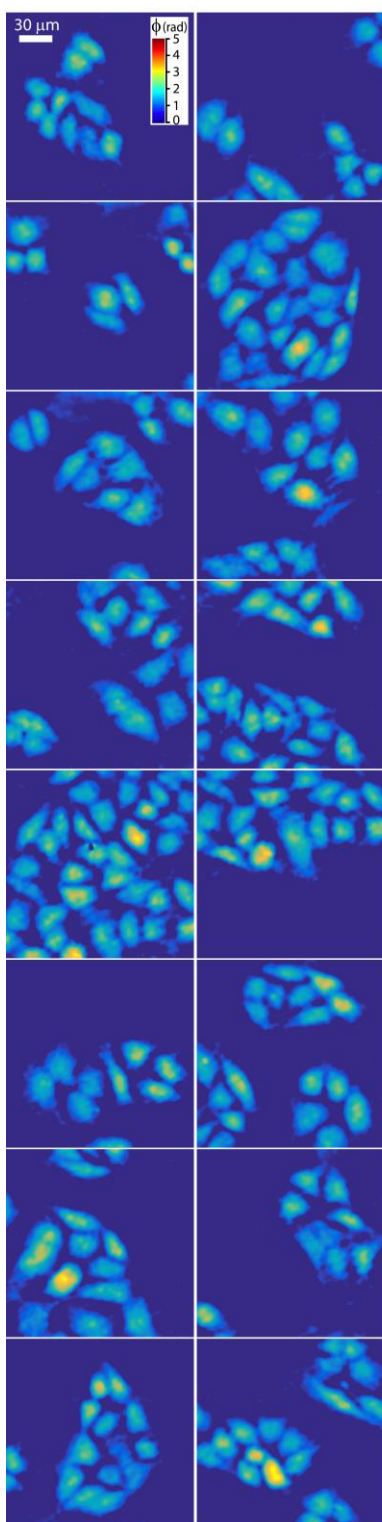

A549 live cells

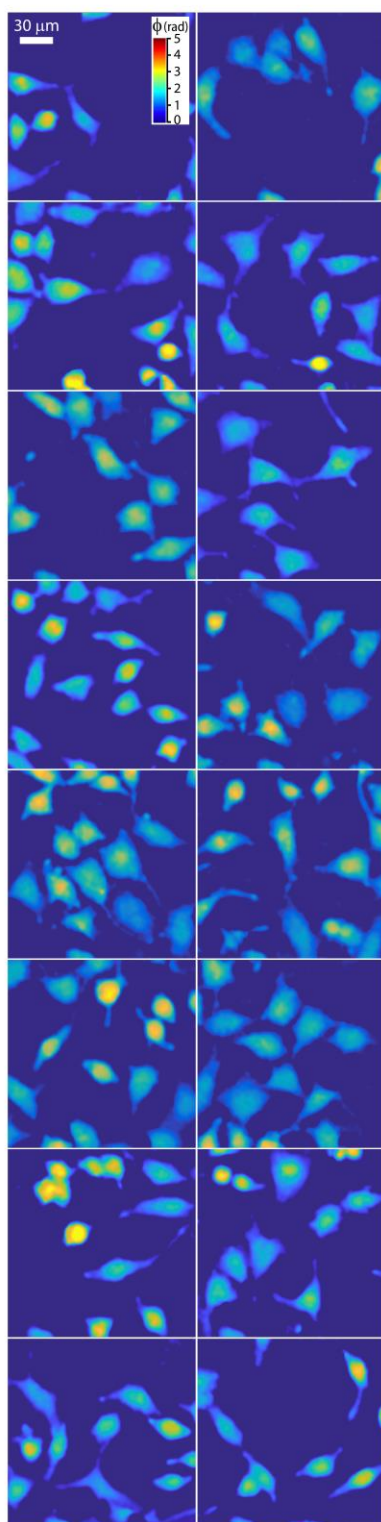

3T3 live cells

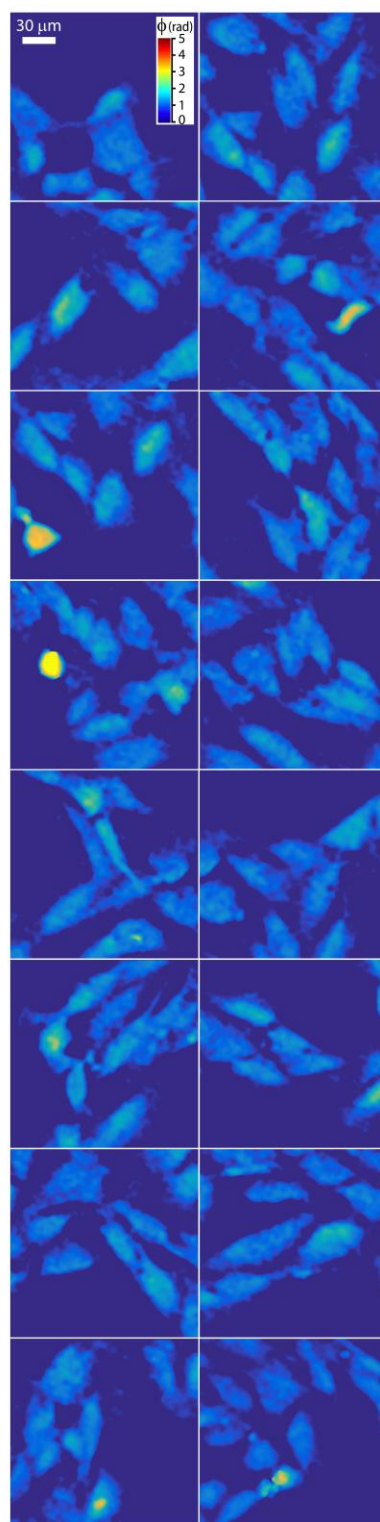

Supplement: Supplementary file 1 [file cells-10-02587-s001.zip › cells-1376145-SI.pdf]
